# 3,5-Bis(4-fluoro­phen­yl)isoxazole

**DOI:** 10.1107/S160053681202171X

**Published:** 2012-05-19

**Authors:** Hoong-Kun Fun, Suhana Arshad, S. Samshuddin, B. Narayana, B. K. Sarojini

**Affiliations:** a X-ray Crystallography Unit, School of Physics, Universiti Sains Malaysia, 11800 USM, Penang, Malaysia; b Department of Studies in Chemistry, Mangalore University, Mangalagangotri 574 199, India; c Department of Chemistry, P. A. College of Engineering, Nadupadavu, Mangalore 574 153, India

## Abstract

In the crystal structure of the title compound, C_15_H_9_F_2_NO, the complete mol­ecule is generated by a crystallographic twofold rotation axis and the O and N atoms of the central isoxazole ring are statistically disordered with equal site occupancies. The terminal benzene rings form a dihedral angle of 24.23 (3)° with the isoxazole ring. The dihedral angle between the benzene rings is 47.39 (2)°. No significant inter­molecular inter­actions are observed.

## Related literature

For the pharmacological activity of isoxazole derivatives, see; Pradeepkumar *et al.* (2011 ▶). For our work on the synthesis of different derivatives of 4,4′-difluoro chalcone, see: Fun *et al.* (2010*a* ▶,*b* ▶). For stability of the temperature controller used in the data collection, see: Cosier & Glazer (1986 ▶).
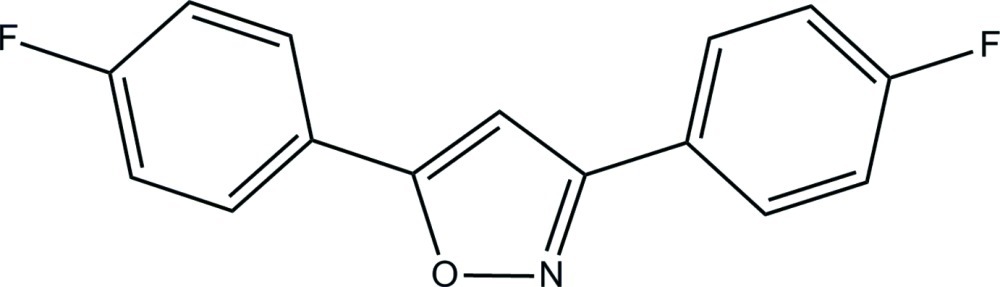


## Experimental

### 

#### Crystal data

C_15_H_9_F_2_NO
*M* *_r_* = 257.23
Monoclinic, 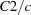

*a* = 27.9097 (4) Å
*b* = 5.7319 (1) Å
*c* = 7.1437 (1) Å
β = 102.473 (1)°
*V* = 1115.84 (3) Å^3^
*Z* = 4
Mo *K*α radiation
μ = 0.12 mm^−1^
*T* = 100 K
0.30 × 0.24 × 0.12 mm

#### Data collection

Bruker SMART APEXII CCD area-detector diffractometer
Absorption correction: multi-scan (*SADABS*; Bruker, 2009 ▶) *T*_min_ = 0.965, *T*_max_ = 0.986
17407 measured reflections
2483 independent reflections
2175 reflections with *I* > 2σ(*I*)
*R*_int_ = 0.024

#### Refinement

*R*[*F* ^2^ > 2σ(*F* ^2^)] = 0.045
*wR*(*F* ^2^) = 0.131
*S* = 1.09
2483 reflections
87 parameters
H-atom parameters constrained
Δρ_max_ = 0.62 e Å^−3^
Δρ_min_ = −0.31 e Å^−3^

### 

Data collection: *APEX2* (Bruker, 2009 ▶); cell refinement: *SAINT* (Bruker, 2009 ▶); data reduction: *SAINT*; program(s) used to solve structure: *SHELXTL* (Sheldrick, 2008 ▶); program(s) used to refine structure: *SHELXTL*; molecular graphics: *SHELXTL*; software used to prepare material for publication: *SHELXTL* and *PLATON* (Spek, 2009 ▶).

## Supplementary Material

Crystal structure: contains datablock(s) global, I. DOI: 10.1107/S160053681202171X/is5139sup1.cif

Structure factors: contains datablock(s) I. DOI: 10.1107/S160053681202171X/is5139Isup2.hkl

Supplementary material file. DOI: 10.1107/S160053681202171X/is5139Isup3.cml

Additional supplementary materials:  crystallographic information; 3D view; checkCIF report

## Figures and Tables

**Fig. 1.**
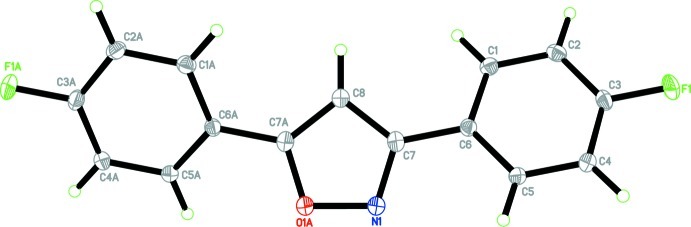
The first disorder component of the title compound, showing 50% probability displacement ellipsoids and the atom-numbering scheme. Atoms with suffix A are generated by symmetry code -x + 1, y, -z + 1/2.

**Fig. 2.**
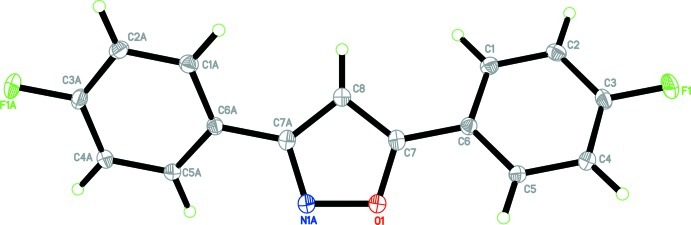
The second disorder component of the title compound, showing 50% probability displacement ellipsoids and the atom-numbering scheme. Atoms with suffix A are generated by symmetry code -x + 1, y, -z + 1/2.

**Fig. 3.**
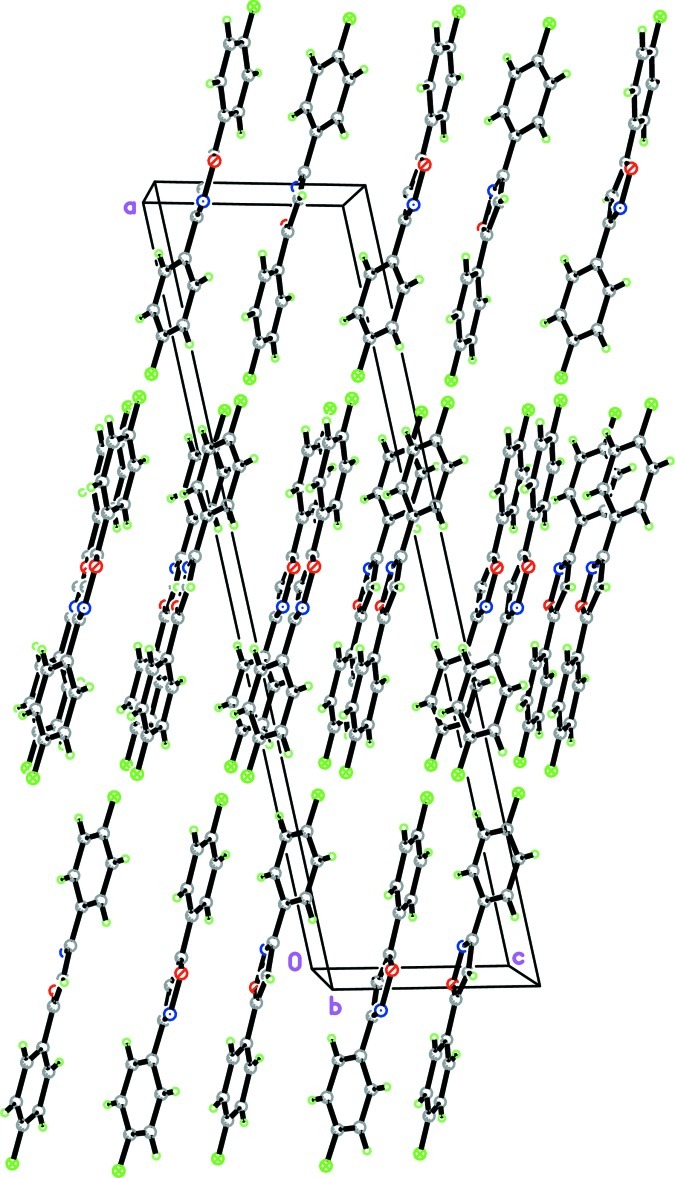
A crystal packing diagram of the title compound, viewed along the b axis.

## supplementary crystallographic information

### Comment

The various pharmacological activities of isoxazole derivatives are well documented (Pradeepkumar *et al.*, 2011). Hence, in view of the importance of isoxazoles and in continuation of our work on synthesis of various derivatives of of 4,4'-difluoro chalcone (Fun *et al.*, 2010*a*,*b*), the title compound was prepared and its crystal structure is reported.

The asymmetric unit of the title molecule (Figs. 1 and 2), C_15_H_9_F_2_NO, contains one half-molecule with the other half of the molecule being generated by a twofold rotation axis (-*x* + 1, *y*, -*z* + 1/2). The crystal structure is disordered with the O1 and the N1 atoms attached at the same position with half occupancies each, forming the central isoxazole ring. The fluoro-substituted benzene rings (C1–C6 & C1A–C6A) make a dihedral angle of 24.23 (3)° with the isoxazole ring (N1/O1A/C7/C7A/C8 or O1/N1A/C7/C7A/C8). The dihedral angle between the fluoro-substituted benzene rings is 47.39 (2)°. The bond lengths and angles are within normal ranges. The crystal packing is shown in Fig. 3. No significant intermolecular interactions were observed.

### Experimental

A solution of 4,4'-difluoro chalcone (2.44 g, 0.01 mol) and hydroxylamine hydrochloride (0.695 g, 0.01 mol) in 25 ml ethanol containing 3 ml of 10% sodium hydroxide solution was refluxed for 12 h. The reaction mixture was cooled and poured into 50 ml ice-cold water. The precipitate formed was collected by filtration and purified by recrystallization from ethanol. The single crystals were grown from a DMF solution by slow evaporation method and yield of the compound was 59%. (*M. p.* 463 K).

### Refinement

The crystal structure is disordered at atom N1 and O1 with refined site of occupancies closed to 0.5. In the final refinement, the ratio was fixed at 0.5: 0.5. All the H atoms were positioned geometrically (C—H = 0.95 Å) and refined using a riding model with *U*_iso_(H) = 1.2*U*_eq_(C). The same atomic coordinates and displacement parameters were used for atom pair O1/N1. Three outliers (2 0 0), (5 1 3) and (9 1 1) were omitted.

### Crystal data

**Table:**

C_15_H_9_F_2_NO	*F*(000) = 528
*M**_r_* = 257.23	*D*_x_ = 1.531 Mg m^−^^3^
Monoclinic, *C*2/*c*	Mo *K*α radiation, λ = 0.71073 Å
Hall symbol: -C 2yc	Cell parameters from 7031 reflections
*a* = 27.9097 (4) Å	θ = 3.0–35.2°
*b* = 5.7319 (1) Å	µ = 0.12 mm^−^^1^
*c* = 7.1437 (1) Å	*T* = 100 K
β = 102.473 (1)°	Block, colourless
*V* = 1115.84 (3) Å^3^	0.30 × 0.24 × 0.12 mm
*Z* = 4	

### Data collection

**Table:**

Bruker SMART APEXII CCD area-detector diffractometer	2483 independent reflections
Radiation source: fine-focus sealed tube	2175 reflections with *I* > 2σ(*I*)
Graphite monochromator	*R*_int_ = 0.024
φ and ω scans	θ_max_ = 35.2°, θ_min_ = 3.0°
Absorption correction: multi-scan (*SADABS*; Bruker, 2009)	*h* = −44→44
*T*_min_ = 0.965, *T*_max_ = 0.986	*k* = −9→9
17407 measured reflections	*l* = −11→11

### Refinement

**Table:**

Refinement on *F*^2^	Primary atom site location: structure-invariant direct methods
Least-squares matrix: full	Secondary atom site location: difference Fourier map
*R*[*F*^2^ > 2σ(*F*^2^)] = 0.045	Hydrogen site location: inferred from neighbouring sites
*wR*(*F*^2^) = 0.131	H-atom parameters constrained
*S* = 1.09	*w* = 1/[σ^2^(*F*_o_^2^) + (0.0728*P*)^2^ + 0.6036*P*] where *P* = (*F*_o_^2^ + 2*F*_c_^2^)/3
2483 reflections	(Δ/σ)_max_ < 0.001
87 parameters	Δρ_max_ = 0.62 e Å^−^^3^
0 restraints	Δρ_min_ = −0.31 e Å^−^^3^

### Special details

**Table:**

Experimental. The crystal was placed in the cold stream of an Oxford Cryosystems Cobra open-flow nitrogen cryostat (Cosier & Glazer, 1986) operating at 100.0 (1) K.
Geometry. All e.s.d.'s (except the e.s.d. in the dihedral angle between two l.s. planes) are estimated using the full covariance matrix. The cell e.s.d.'s are taken into account individually in the estimation of e.s.d.'s in distances, angles and torsion angles; correlations between e.s.d.'s in cell parameters are only used when they are defined by crystal symmetry. An approximate (isotropic) treatment of cell e.s.d.'s is used for estimating e.s.d.'s involving l.s. planes.
Refinement. Refinement of *F*^2^ against ALL reflections. The weighted *R*-factor *wR* and goodness of fit *S* are based on *F*^2^, conventional *R*-factors *R* are based on *F*, with *F* set to zero for negative *F*^2^. The threshold expression of *F*^2^ > σ(*F*^2^) is used only for calculating *R*-factors(gt) *etc*. and is not relevant to the choice of reflections for refinement. *R*-factors based on *F*^2^ are statistically about twice as large as those based on *F*, and *R*- factors based on ALL data will be even larger.

### Fractional atomic coordinates and isotropic or equivalent isotropic displacement parameters (Å^2^)

**Table:**

	*x*	*y*	*z*	*U*_iso_*/*U*_eq_	Occ. (<1)
F1	0.26779 (2)	0.15964 (11)	−0.19266 (9)	0.02342 (15)	
O1	0.47501 (2)	0.57010 (12)	0.20224 (10)	0.01759 (15)	0.50
N1	0.47501 (2)	0.57010 (12)	0.20224 (10)	0.01759 (15)	0.50
C1	0.39839 (3)	0.08546 (14)	−0.01408 (11)	0.01434 (15)	
H1A	0.4233	−0.0272	−0.0145	0.017*	
C2	0.35018 (3)	0.03736 (14)	−0.10665 (11)	0.01517 (15)	
H2A	0.3419	−0.1066	−0.1714	0.018*	
C3	0.31471 (3)	0.20453 (15)	−0.10192 (11)	0.01495 (15)	
C4	0.32494 (3)	0.41685 (14)	−0.00912 (12)	0.01460 (15)	
H4A	0.2997	0.5274	−0.0076	0.018*	
C5	0.37327 (3)	0.46326 (13)	0.08167 (11)	0.01285 (14)	
H5A	0.3813	0.6079	0.1457	0.015*	
C6	0.41029 (3)	0.29891 (13)	0.07965 (11)	0.01230 (14)	
C7	0.46118 (3)	0.34794 (14)	0.17574 (12)	0.01458 (15)	
C8	0.5000	0.1978 (2)	0.2500	0.01459 (19)	
H8A	0.5000	0.0321	0.2500	0.018*	

### Atomic displacement parameters (Å^2^)

**Table:**

	*U*^11^	*U*^22^	*U*^33^	*U*^12^	*U*^13^	*U*^23^
F1	0.0136 (2)	0.0260 (3)	0.0270 (3)	−0.00518 (19)	−0.0038 (2)	−0.0017 (2)
O1	0.0133 (3)	0.0143 (3)	0.0232 (3)	0.0005 (2)	−0.0006 (2)	−0.0005 (2)
N1	0.0133 (3)	0.0143 (3)	0.0232 (3)	0.0005 (2)	−0.0006 (2)	−0.0005 (2)
C1	0.0150 (3)	0.0134 (3)	0.0145 (3)	0.0011 (2)	0.0030 (2)	−0.0003 (2)
C2	0.0175 (3)	0.0129 (3)	0.0144 (3)	−0.0023 (2)	0.0019 (2)	−0.0012 (2)
C3	0.0123 (3)	0.0169 (3)	0.0144 (3)	−0.0033 (2)	0.0001 (2)	0.0006 (2)
C4	0.0121 (3)	0.0146 (3)	0.0163 (3)	0.0012 (2)	0.0014 (2)	0.0008 (2)
C5	0.0126 (3)	0.0119 (3)	0.0136 (3)	0.0002 (2)	0.0018 (2)	−0.0004 (2)
C6	0.0115 (3)	0.0129 (3)	0.0121 (3)	0.0002 (2)	0.0016 (2)	0.0005 (2)
C7	0.0121 (3)	0.0170 (3)	0.0141 (3)	−0.0007 (2)	0.0020 (2)	0.0000 (2)
C8	0.0128 (4)	0.0139 (4)	0.0164 (4)	0.000	0.0016 (3)	0.000

### Geometric parameters (Å, º)

**Table:**

F1—C3	1.3546 (9)	C4—C5	1.3903 (10)
O1—C7	1.3322 (10)	C4—H4A	0.9500
O1—N1^i^	1.4145 (13)	C5—C6	1.4006 (11)
C1—C2	1.3926 (11)	C5—H5A	0.9500
C1—C6	1.3998 (11)	C6—C7	1.4650 (11)
C1—H1A	0.9500	C7—C8	1.3955 (10)
C2—C3	1.3833 (12)	C8—C7^i^	1.3955 (10)
C2—H2A	0.9500	C8—H8A	0.9500
C3—C4	1.3858 (12)		
			
C7—O1—N1^i^	107.08 (4)	C5—C4—H4A	121.0
C7—O1—O1^i^	107.08 (4)	C4—C5—C6	120.66 (7)
C2—C1—C6	120.36 (7)	C4—C5—H5A	119.7
C2—C1—H1A	119.8	C6—C5—H5A	119.7
C6—C1—H1A	119.8	C1—C6—C5	119.56 (7)
C3—C2—C1	118.31 (7)	C1—C6—C7	119.85 (7)
C3—C2—H2A	120.8	C5—C6—C7	120.58 (7)
C1—C2—H2A	120.8	O1—C7—C8	110.98 (7)
F1—C3—C2	118.66 (7)	O1—C7—C6	118.14 (7)
F1—C3—C4	118.28 (7)	C8—C7—C6	130.87 (8)
C2—C3—C4	123.06 (7)	C7—C8—C7^i^	103.86 (10)
C3—C4—C5	118.04 (7)	C7—C8—H8A	128.1
C3—C4—H4A	121.0	C7^i^—C8—H8A	128.1
			
C6—C1—C2—C3	−0.49 (12)	N1^i^—O1—C7—C8	0.05 (10)
C1—C2—C3—F1	179.59 (7)	O1^i^—O1—C7—C8	0.05 (10)
C1—C2—C3—C4	−0.16 (12)	N1^i^—O1—C7—C6	179.64 (8)
F1—C3—C4—C5	−179.18 (7)	O1^i^—O1—C7—C6	179.64 (8)
C2—C3—C4—C5	0.57 (12)	C1—C6—C7—O1	156.29 (8)
C3—C4—C5—C6	−0.34 (12)	C5—C6—C7—O1	−24.13 (11)
C2—C1—C6—C5	0.71 (12)	C1—C6—C7—C8	−24.21 (12)
C2—C1—C6—C7	−179.70 (7)	C5—C6—C7—C8	155.37 (7)
C4—C5—C6—C1	−0.29 (12)	O1—C7—C8—C7^i^	−0.02 (4)
C4—C5—C6—C7	−179.87 (7)	C6—C7—C8—C7^i^	−179.55 (10)

Symmetry code: (i) −*x*+1, *y*, −*z*+1/2.
